# STAT4 Is Largely Dispensable for Systemic Lupus Erythematosus–like Autoimmune- and Foreign Antigen–Driven Antibody-Forming Cell, Germinal Center, and Follicular Th Cell Responses

**DOI:** 10.4049/immunohorizons.2000111

**Published:** 2021-01-14

**Authors:** Adam J. Fike, Sathi Babu Chodisetti, Kristen N. Bricker, Nicholas M. Choi, Zissis C. Chroneos, Mark H. Kaplan, Ziaur S. M. Rahman

**Affiliations:** * Department of Microbiology and Immunology, Pennsylvania State University College of Medicine, Hershey, PA 17033; † Department of Pediatrics, Pennsylvania State University College of Medicine, Hershey, PA 17033; ‡ Pulmonary Immunology and Physiology Laboratory, Pennsylvania State University College of Medicine, Hershey, PA 17033; § Department of Microbiology and Immunology, Indiana University School of Medicine, Indianapolis, IN 46202

## Abstract

Genome-wide association studies identified variants in the transcription factor *STAT4* gene and several other genes in the STAT4 signaling pathway, such as *IL12A*, *IL12B*, *JAK2*, and *TYK2*, which are associated with an increased risk of developing systemic lupus erythematosus (SLE) and other autoimmune diseases. Consistent with the genome-wide association studies data, STAT4 was shown to play an important role in autoimmune responses and autoimmunity development in SLE mouse models. Despite such important role for STAT4 in SLE development in mice and humans, little is known whether and how STAT4 may regulate extrafollicular Ab-forming cell (AFC) and follicular germinal center (GC) responses, two major pathways of autoreactive B cell development and autoantibody production. To our surprise, we found STAT4 to be largely dispensable for promoting autoimmune AFC and GC responses in various autoimmune- and SLE-prone mouse models, which strongly correlated with autoantibody production, and immune complex deposition and immune cell infiltration in the kidney. We further observed that STAT4 deficiency had no effects on AFC, GC, and Ag-specific Ab responses during protein Ag immunization or influenza virus infection. Additionally, CD4^+^ effector and follicular Th cell responses in autoimmune- and SLE-prone mice and protein Ag–immunized and influenza virus–infected mice were intact in the absence of STAT4. Together, our data demonstrate a largely dispensable role for STAT4 in AFC, GC, and Ab responses in SLE mouse models and in certain foreign Ag–driven responses. *ImmunoHorizons*, 2021, 5: 2–15.

## INTRODUCTION

Although B cell responses are protective during microbial infection or foreign Ag challenge, they also can be pathogenic in promoting the development of autoimmune diseases like systemic lupus erythematosus (SLE) (1–3). Understanding differential mechanisms that control protective and pathogenic B cell responses would help target B cells for treating autoimmune diseases or vaccine development and is the focus of many current research efforts. B cell responses are divided into two major branches, germinal center (GC) and extrafollicular Ab-forming cell (AFC) pathways (4, 5). Canonically, the GC response is a slow process that generates high-affinity, class-switched Ab and long-lived plasma cells and memory B cells under the selective regulation of T follicular helper cells (Tfh) (6, 7). Conversely, the more rapid extrafollicular AFC response is short-lived, can be T cell dependent, in which help is provided by CD4^+^ effector T (Th) cells or T cell independent, and generates low-affinity IgM and class-switched Abs (8–10). Developing an understanding of the regulation of AFC and GC pathways is critical in identifying the mechanisms of autoimmune and pathogen-driven B cell responses.

STAT4 is a transcription factor that is activated via classical JAK/STAT signaling following stimulation by various cytokines such as IL-12, IL-23, and/or type I IFN (11–13). Numerous in vitro studies in T cells from humans and mice have highlighted a critical role for STAT4 in the commitment of activated CD4^+^ T cells toward a Tbet-expressing and IFN-γ–producing Th1 lineage in an IL-12–dependent manner (11, 12, 14–17). Specifically, previous studies demonstrated that early Th1 commitment is mediated through synergistic signaling of the TCR paired with IFN-γ signaling, whereas late commitment of Th1 is mediated through the IL-12–STAT4 axis (18). Another in vitro study indicated that IL-12 signaling via STAT4 induces IL-21 and Bcl6 genes in T cells, promoting the differentiation of T cells with features of Tfh producing both IL-21 and IFN-γ (19). In addition, STAT4 deficiency was recently shown to cause a moderate reduction in IFN-γ production by Tfh, although it did not result in decreased Tfh populations during acute lymphocytic choriomeningitis virus infection (20). Early studies also demonstrated that Th1 cytokines are associated with IgG2a Ab production, whereas STAT4 deficiency is associated with a reduction in IFN-γ production, increases in Th2-associated cytokines, and a switch to IgG1 Ab production in immunization and infection systems (21–23). However, the exact role of STAT4 in the regulation of foreign Ag–driven AFC and GC responses during immunization or infection is not clear.

We and others previously demonstrated that systemic and B cell–intrinsic IFN-γR, STAT1, and Tbet expression are critical in the regulation of autoimmune AFC and GC responses and the development of murine SLE disease (4, 24, 25). Numerous genome-wide association studies (GWAS) on SLE patients have identified variants in the *STAT4* gene that are associated with an increased risk of developing SLE and other autoimmune diseases (26–32). STAT4 regulates T cell inflammatory cytokine (IFN-γ, IL-17, and IL-21) production that promotes SLE pathogenesis in mice and humans (33). Two previous studies investigated the role of STAT4 in autoimmunity development in SLE mouse models that showed some consistent but also conflicting findings (34, 35). Although both studies reported reduced autoantibody responses, one study showed exacerbated lupus nephritis and mortality in the absence of STAT4 in NZM2328 SLE-prone mice (35), whereas the other study in SLE1,2,3-triple congenic mice deficient in STAT4 revealed ameliorated lupus nephritis and improved survival (34). Neither study, however, directly examined the effect of STAT4 deficiency on the development of autoimmune AFC and GC B cell responses. Further, the discrepancy in the role of STAT4 in kidney pathology in these two models remains unclear.

In this study, we investigated the role of STAT4 in the regulation of AFC and GC responses in the context of autoimmune and foreign Ag–driven responses. We examined the autoimmune responses including AFC, GC, and CD4^+^ T cell responses in the absence of STAT4 in autoimmune-prone Sle1b mice (36, 37); TLR7-induced SLE-prone B6.Sle1b (Sle1b) mice (38); and spontaneous (Spt), SLE-prone FcyRIIB^−/−^ mouse strain (39, 40). To our surprise, we found that the STAT4 deficiency resulted in no significant attenuation to autoimmune AFC, GC, and Th cell responses in any of these models, which strongly correlated with autoantibody production and kidney immune complex (IC) deposition and immune cell infiltration. We further observed that Tbet expression in CD4^+^ Th1 T cell and IFN-γ transcript level in the spleen were intact in the absence of STAT4. To determine the role of STAT4 in foreign Ag–driven AFC, GC, and Th cell responses, we examined these responses in STAT4^−/−^ mice after T-dependent immunization (4-hydroxy-3-nitrophenol–keyhole limpet hemocyanin [NP-KLH]) and influenza viral infection. Again, we found no significant effects of STAT4 deficiency on AFC, GC, and Th cell responses and Ag-specific Ab production either during NP-KLH immunization or influenza viral infection. Together, our findings suggest that STAT4 is dispensable for promoting both autoimmune and antipathogen AFC, GC, and Tfh responses in these autoimmune- or SLE-prone and certain exogenous Ag–immunized or –infected mice.

## MATERIALS AND METHODS

### Mice

C57BL/6J (B6) and C57BL/6J-*Stat4*^*em3Adiuj*^/J (STAT4^−/−^) mice were originally purchased from The Jackson Laboratory and bred in-house. B6.129S4-*Fcgr2b*^*tm1TtK*^ N12 (FcyRIIB^−/−^) mice were originally purchased from Taconic Biosciences and were bred in-house. B6.Sle1b (Sle1b) mice (congenic for the Sle1b sublocus) were previously described (36). Crossing STAT4^−/−^mice to autoimmune-prone strains was performed in-house. All animal studies were performed within American Association for the Accreditation of Laboratory Animal Care–certified barrier facility within Pennsylvania State University College of Medicine Hershey Medical Center. Mice were housed under specific pathogen-free conditions. All animal work was performed in accordance with protocols approved by our Institutional Animal Care and Use Committee.

### Flow cytometry

Splenic, lung, and kidney tissues were processed into single-cell suspensions and stained using the following Abs: B220-BV605 (RA3–6B2), CD4-AF700 (RMP4–5), CD44-APC (IM7), CD62L–PE-Cy7 (MEL-14), PD1-PE (29F.1A12), CD138–PE-Cy7(281–2), TACI-PE (8F10), CD19-BV605 (6D5) (all from BioLegend), GL7-FITC (GL-7), CD95–PE-Cy7, CXCR5–biotin (2G8), and B220–Pacific Blue (RA3–6B2) (BD Biosciences). CXCR5 staining was performed at room temperature for 30 min. All other staining was performed at 4°C to prevent internalization. Prior to surface staining, all cells were stained using the fixable viability dye eFluor 780 (Invitrogen). Intracellular Tbet staining was performed using the FoxP3 transcription factor buffer set (Thermo Fisher Scientific) according to manufacturer’s guidelines and Tbet-APC (4B10) (BioLegend). Stained cells were analyzed on a BD LSR II flow cytometer using FACSDiva software (BD Biosciences). Flow cytometry data were analyzed using FlowJo software (Tree Star).

### Immunofluorescence microscopy and Hep-2 antinuclear Ab profiling

For splenic or kidney microscopy, tissues were first embedded in OCT compound and snap frozen over liquid nitrogen. Five-μm sections were cut on a cryostat, mounted on ColorFrost Plus Microscope Slides (Thermo Fisher Scientific) and fixed in cold acetone for 20 min. For visualizing GCs within spleens, sections were stained with the following Abs: GL7-FITC (GL-7; BD Biosciences), CD4-PE (GK1.2; BioLegend), and IgD-APC (11–26c2a; BD Biosciences). Kidney sections were stained with C3-FITC (Immunology Consultant Laboratory) and Anti-IgG-PE (Abcam) for the detection of IC depositions. For antinuclear Ab (ANA) seropositivity, Hep-2 slides were incubated with mouse serum at a 1:50 dilution. ANAs were then detected using an FITC-rat anti-mouse κ Ab (H139–52.1; Southern Biotech). Images of stained tissue sections and ANA slides were performed using a Leica DM4000 fluorescence microscope and analyzed using a Leica Application Suite Advanced Fluorescence software (Leica Micro-systems). GC measurements represent a random selection of GL-7^+^ staining across the splenic tissue section in which total GC areas (square micrometers) were measured using the Leica Application Suite Advanced Fluorescence quantification tool. C3 deposition within the kidney was quantified as previously reported (41).

### ELISAs

Serum ANA detection was performed as previously described (4, 42). In short, ELISA plates were coated with salmon sperm dsDNA (Invitrogen), nucleosome (histone from Sigma Aldrich on a layer of dsDNA coating), or small ribonucleoprotein (SmRNP) (Arotec Diagnostics). Plates were blocked with 5% FBS, and serum was added with a starting dilution of 1:50, followed by a 1:2 serial dilution for the remainder of the plate. For the detection of NP-specific Abs, ELISA plates were coated with 10 μg/ml of NP_4_-BSA or NP_29_-BSA and blocked with 5% BSA. Serum was added at a dilution starting at 1:1000, followed by serial dilution. For measuring influenza virus–specific serum Abs ELISAs plates were coated with 175 ng of influenza virus and blocked with 5% BSA. Serum was added starting at a 1:25 dilution, followed by serial dilution. Total Abs were detected by coating plates with goat anti-mouse IgM (Thermo Fisher Scientific) or anti-IgG (Invitrogen) at 10 μg/ml and blocked with 5% BSA. Abs were then detected with the following biotinylated Abs: goat anti-mouse IgM (Jackson ImmunoResearch Laboratories), goat anti-mouse IgG (Jackson ImmunoResearch Laboratories), Goat Anti-Mouse IgG1 (SouthernBiotech), or Goat Anti-Mouse IgG2c (SouthernBiotech), followed by streptavidin–alkaline phosphatase (Vector Laboratories). Plates were developed using *p*-nitrophenyl phosphate (disodium salt) (Thermo Fisher Scientific) substrates for alkaline phosphatase and read at λ405 nm on Synergy H1 (BioTek Instruments).

### Quantitative RT-PCR

For quantitative RT-PCR analysis, RNA was isolated from splenocytes via TRIzol (Invitrogen) chloroform precipitation according to manufacturer’s instructions. Precipitated RNA was further purified via the Qiagen RNeasy kit according to manufacturer’s instructions (QIAGEN). RNA quality and quantity were measured via nanodrop prior to cDNA synthesis (Thermo Fisher Scientific). cDNA was generated using the High Capacity cDNA Kit according to manufacturer’s instructions. *Ifng*, *Stat1*, and *Il12p35* transcript levels were assessed using the following primer sequences and SYBR Green Master Mix (Thermo Fisher Scientific): *Il12p35* forward (5′-CACAAGAACGAGAGTTGCCTGGCT-3′) reverse (5′-GGTCTGCTTCTCCCACAGGAGGTT-3′), *Ifng* forward (5′-CGGCACAGTCATTGAAAGCC-3′) reverse (5′-TGCATCCTTT TTCGCCTTGC-3′), and Stat1 PrimeTime (Mm.PT.58.23792152) (Integrated DNA Technologies). Transcript levels were normalized against a housekeeping gene (β-actin), and fold change was calculated via the 2^(ΔΔCt)^ method.

### Imiquimod treatment

Mice were treated epicutaneously with imiquimod (Imq) as previously reported (4). In short, 5% Imq cream (Perrigo) was applied to the ears three times a week for 8 or 12 wk based on the experimental design. Imq treatment does not result in visible cutaneous lesions on the ears as described (4).

### NP-KLH immunization and influenza viral infection

Eight- to ten-week-old mice were immunized with 200 μg/mouse of NP-KLH (Biosearch Technologies) i.p. in CFA (Sigma-Aldrich). Mice received a booster immunization on day 7 postimmunization with 100 μg NP-KLH suspended in IFA via i.p. injection. Spleens were harvested and analyzed on day 14 after initial immunization.

Mice were infected intranasally with 1000 fluorescent focus units of influenza virus H1N1 strain (A/Puerto Rico/8/34) in a 40-μl volume as described (43). Mice were anesthetized with inhaled isoflurane before intranasal inoculations. Weight loss was tracked by daily measurements. Lungs and spleens were harvested at day 14 postinfection for various analysis.

### Isolation of pulmonary lymphocytes

For isolation of pulmonary lymphocytes following influenza viral infection, lungs were harvested from individual mice and minced into small pieces. The lung tissue was then digested for 2 h at 37°C with 3.0 mg/ml collagenase A (Sigma-Aldrich) and 0.15 μg/ml DNase I (Sigma-Aldrich) in RPMI 1640 (Corning). Digested tissue was then run through a 40-μM cell strainer (Falcon). The strainer was washed using the complete RPMI 1640 described above. Cells were counted via trypan blue exclusion with a Motic ae31 light microscope (Motic). Cells were then stained as described above for flow cytometry.

### Statistical analysis

Normality of datasets was first assessed by D’Agostino–Pearson test for normality. Statistical analysis was then performed by Mann–Whitney, Student *t* test, or one-way ANOVA with multiple-comparisons follow-up based on the degree of normality. The *p* values were as follows: *p < 0.05, **p < 0.01, ***p < 0.001, and ****p < 0.0001. Statistical analysis was performed in GraphPad Prism version 6 (GraphPad Software, La Jolla, CA).

## RESULTS

### STAT4 is not required for autoimmune B cell responses in SLE-prone FcyRIIB^−/−^ mice

**FIGURE 1.**
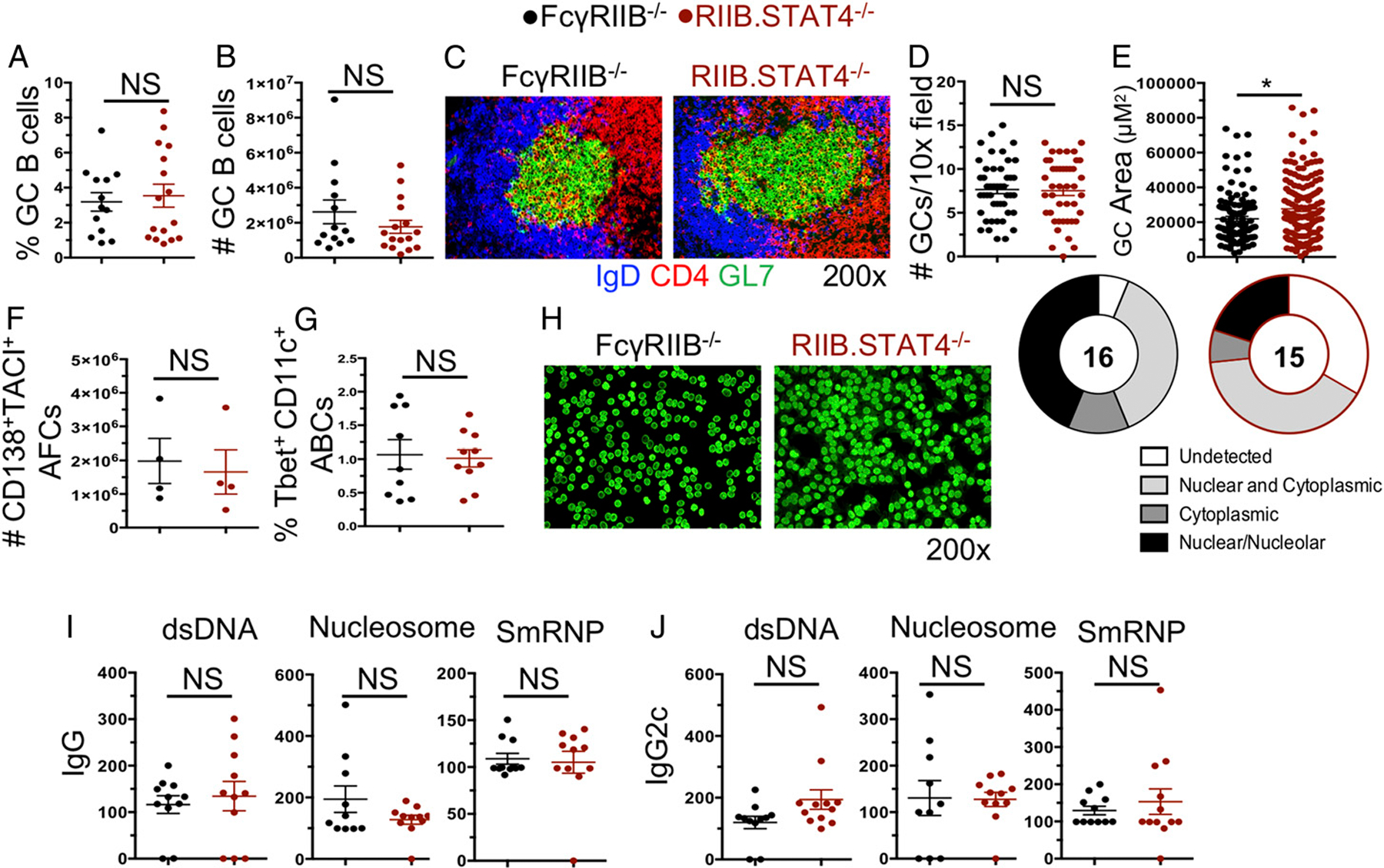
STAT4 is not required for autoimmune B cell responses in FcγRIIB^−/−^ SLE model. Autoimmune B cell responses were assessed in 4-mo-old RIIB^−/−^.STAT4^−/−^ and FcγRIIB^−/−^ control mice. (**A**) Frequency and (**B**) number of GC B cells (CD95^+^GL-7^+^) of total B220^+^ B cells in the spleens assessed by flow cytometry. (**C**) Representative IF images of GCs within the spleen at original magnification ×200. Quantification of the number (**D**) and size (**E**) of GCs per 10× field within the spleens of indicated mice. (**F**) Number of total IgD^−^CD138^+^TACI^+^ AFCs in the spleen. (**G**) Frequency of Tbet^+^CD11c^+^ age-associated B cells (ABCs) in the spleens of indicated mice. (**H**) Representative Hep-2 slides stained with anti-κ Ab at (original magnification ×200) showing ANA reactivity with various patterns shown in the pie chart in which the number in center indicates the number of animals assessed. IgG-specific (**I**) and IgG2c-specific (**J**) serum Ab titers against dsDNA, nucleosomes, and SmRNP in these mice. Each point is an individual mouse. *n* = 5–16 mice per genotype, two to four independent experiments. *p < 0.05.

To determine the role of STAT4 in SLE-associated Spt-AFC and Spt-GC responses and SLE-like autoimmunity development, we crossed STAT4^−/−^ mice to the SLE-prone FcγRIIB^−/−^ mouse strain (designated RIIB^−/−^.STAT4^−/−^). FcγRIIB^−/−^ mice originally generated on 129 background and then backcrossed for many generations to B6 develop SLE-like disease (39–41) mediated by FcγRIIB deficiency and a remaining 0.8% genome derived from 129 including SLE-associated SLAM family genes (41). FcγRIIB^−/−^ mice develop rampant systemic autoimmunity with readily detectable IC deposition within the kidney as early as 3–4 mo of age (41). We first determined the Spt-GC response at 4 mo of age by flow cytometry within the spleen and found no significant difference in the frequency or number of GC B cells between RIIB^−/−^.STAT4^−/−^ and FcγRIIB^−/−^ control mice (Fig. 1A, 1B). We further examined the size and number of GCs in the spleen by immunofluorescence microscopy and found that although the number of GCs within the spleen were similar, RIIB^−/−^STAT4^−/−^ mice had a surprising increase in the size of the GCs (Fig. 1C–E). We then analyzed the Spt-AFC responses in the spleen and found no significant difference in the percentage (data not shown) or number of CD138^+^TACI^+^ AFCs between the strains (Fig. 1F). We also did not find any effect of STAT4 deficiency on the development of Tbet^+^CD11c^+^ age-associated B cells (Fig. 1G), which are associated with SLE-like autoimmune responses and is highly induced following stimulation with Th1-cytokine IFN-γ (44).

We next examined the role of STAT4 deficiency in the ability of FcγRIIB^−/−^ mice to generate autoantibodies. We first determined the ANA seropositivity and found no significant difference between FcγRIIB^−/−^ and RIIB^−/−^.STAT4^−/−^ mice, although some differences in staining patterns were observed (Fig. 1H). We then measured serum IgG and IgG2c autoantibody titers directed against dsDNA, nucleosome, and SmRNP and found no significant impact of STAT4 deficiency on IgG (Fig. 1I) or IgG2c (Fig. 1J) autoantibody titers. Given these surprising findings, we regenotyped mice to demonstrate that RIIB^−/−^.STAT4^−/−^ mice were indeed deficient in STAT4 (Supplemental Fig. 1A), and no STAT4 protein was detected in B cells deficient in STAT4 in the presence of various cytokine stimulations that activate STAT4 (Supplemental Fig. 1B). Together, these data demonstrate that STAT4 deficiency does not attenuate autoimmune B cell responses in the FcγRIIB^−/−^ model.

### CD4^+^ T cell responses unaltered in FcγRIIB^−/−^ mice deficient in STAT4

**FIGURE 2.**
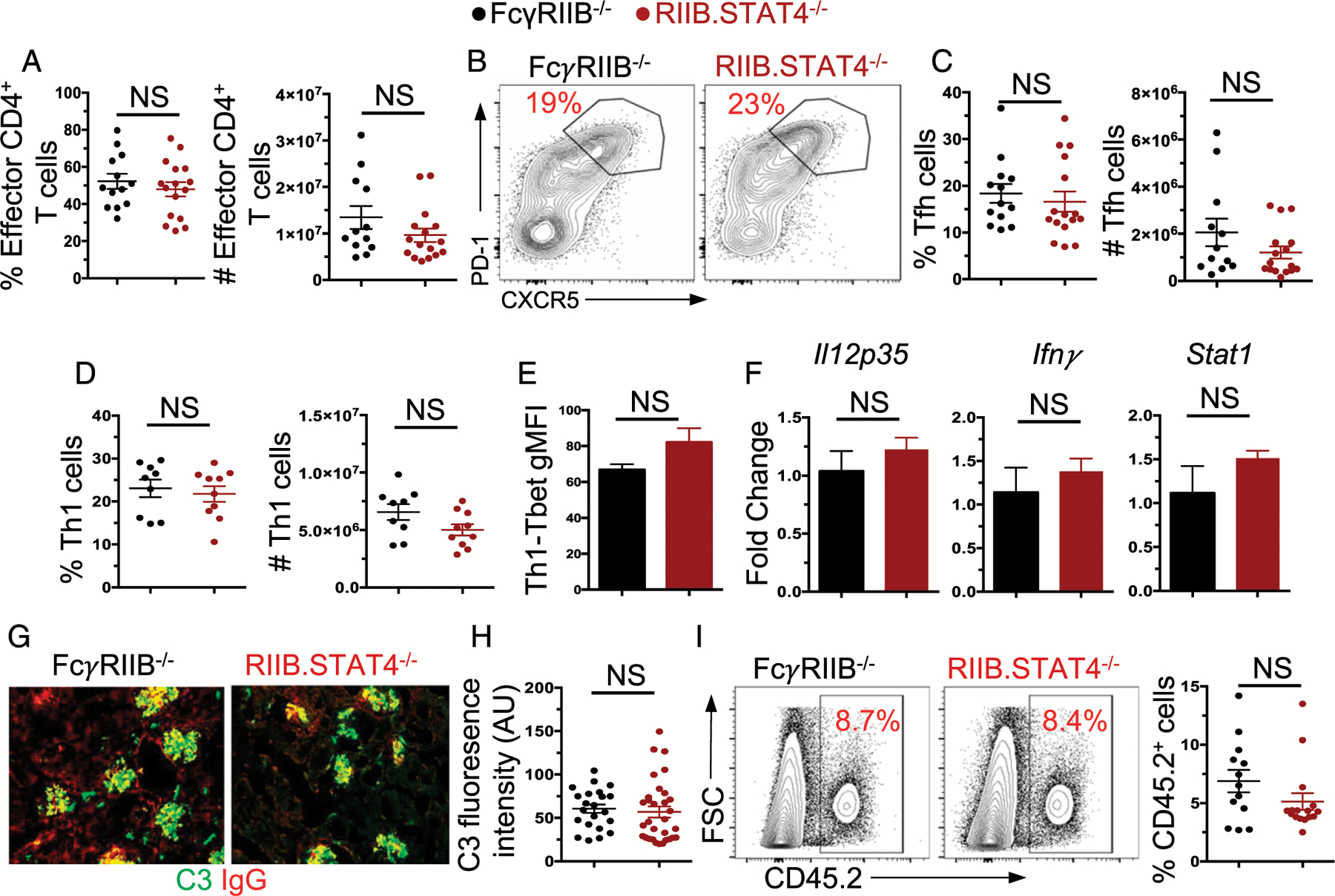
CD4^+^ Th cell responses and SLE kidney manifestations are intact in FcγRIIB^−/−^ mice deficient in STAT4. CD4^+^ T cell responses were assessed in the spleens of 4-mo-old RIIB^−/−^.STAT4^−/−^ and FcγRIIB^−/−^ control mice. (**A**) Frequency and number of CD4^+^CD44^+^CD62L^−^ effector T cells in the spleen of indicated mice. (**B**) Representative flow plots of CD4^+^CD44^+^CD62L^−^PD-1^+^CXCR5^+^ Tfh. (**C**) Frequency and number of Tfh in the spleen. (**D**) Frequency and number of CD4^+^PSGL-1^+^Ly-6C^−^ Th1 cells within the spleen. (**E**) Geometric mean fluorescent intensity (gMFI) of Tbet expression in Th1 cells. (**F**) RT-PCR assessment of *Il12p35*, *Ifng*, and *Stat1* transcript levels in splenocytes. (**G**) Representative images (original magnification ×200) of IC deposition within the kidneys of indicated mice and (**H**) quantification of C3 fluorescence intensity from 20 randomly selected glomeruli. (**I**) Representative FACS plots and frequency of CD45.2^+^ infiltrates into the kidney. Each dot point is an individual mouse. These data were obtained from 9 to 16 mice per genotype and two to four independent experiments. NS, not significant.

Previous in vitro studies highlighted the role of STAT4 in regulating CD4^+^ T cell differentiation into Th1 lineage cells (11, 12, 45, 46). We assessed whether STAT4 deficiency altered the development of various T cell populations during autoimmune responses in FcγRIIB^−/−^ mice. Consistent with our findings of B cell responses, the T cell compartment appeared largely unaltered in the absence of STAT4. Specifically, the frequency and number of CD4^+^CD44^+^CD62L^−^ effector T cells were not impacted by the loss of STAT4 (Fig. 2A) in FcγRIIB^−/−^ mice. Further, the frequency and number of CD4^+^CD44^+^CD62L^−^PD-1^+^CXCR5^+^ Tfh (Fig. 2B, 2C) were also not different between FcγRIIB^−/−^ and RIIB^−/−^.STAT4^−/−^ mice. To further rule out an effect of STAT4 on Th1 cell development, we assessed the percentage and number of CD4^+^PSGL-1^+^Ly-6c^+^ T cells that have previously been characterized as CD4^+^ Th1 cells (20) and found no difference between the strains (Fig. 2D). We then measured Tbet expression in CD4^+^ Th1 cells by flow cytometry and found no difference between the two strains (Fig. 2E). We also assessed the transcript levels of *Il12p35*, *Ifnγ*, and *Stat1* in splenocytes from FcγRIIB^−/−^ and RIIB^−/−^.STAT4^−/−^ mice at 4 moof age, which were found to be unaltered by the STAT4 deficiency (Fig. 2F). Together, these findings demonstrate that despite the significant implications for STAT4 in the regulation of a Th1-driven CD4^+^ T cell response, STAT4 deficiency does not impact the differentiation of this and other populations of CD4^+^ T cells during autoimmune responses in FcγRIIB^−/−^ mice.

### STAT4 deficiency does not affect IC deposition and immune cell infiltration in the kidney of FcγRIIB^−/−^ mice

Despite our findings that STAT4 does not overtly affect Spt-AFC and -GC and various CD4^+^ effector T cell responses, we investigated whether STAT4 may play a role in the hallmark kidneymanifestations of SLE-prone mice, particularly given the previous observations showing acceleration of kidney disease in one model (35) and amelioration in the other SLE model deficient in STAT4 (34). We harvested the kidneys from FcγRIIB^−/−^ and RIIB^−/−^.STAT4^−/−^ mice and assessed IgG and C3 IC deposition within the kidneys. We found that IC deposition was largely intact in the absence of STAT4 (Fig. 2G, 2H). We further assessed immune cell infiltrates in the kidney by flow cytometry and found no difference between the genotypes (Fig. 2I). We also determined Spt-GC and -AFC and Th cell responses, autoantibody titers, and kidney IC deposition in 9-mo-old FcγRIIB^−/−^ and RIIB^−/−^.STAT4^−/−^ mice and found similar results (data not shown). Together, these data indicate that STAT4 is largely dispensable for mounting autoimmune AFC, GC, and T cell responses and autoimmunity development in SLE-prone FcγRIIB^−/−^ mice.

### STAT4 is not required for Spt-GC and -AFC and Th cell responses in Sle1b mice

**FIGURE 3.**
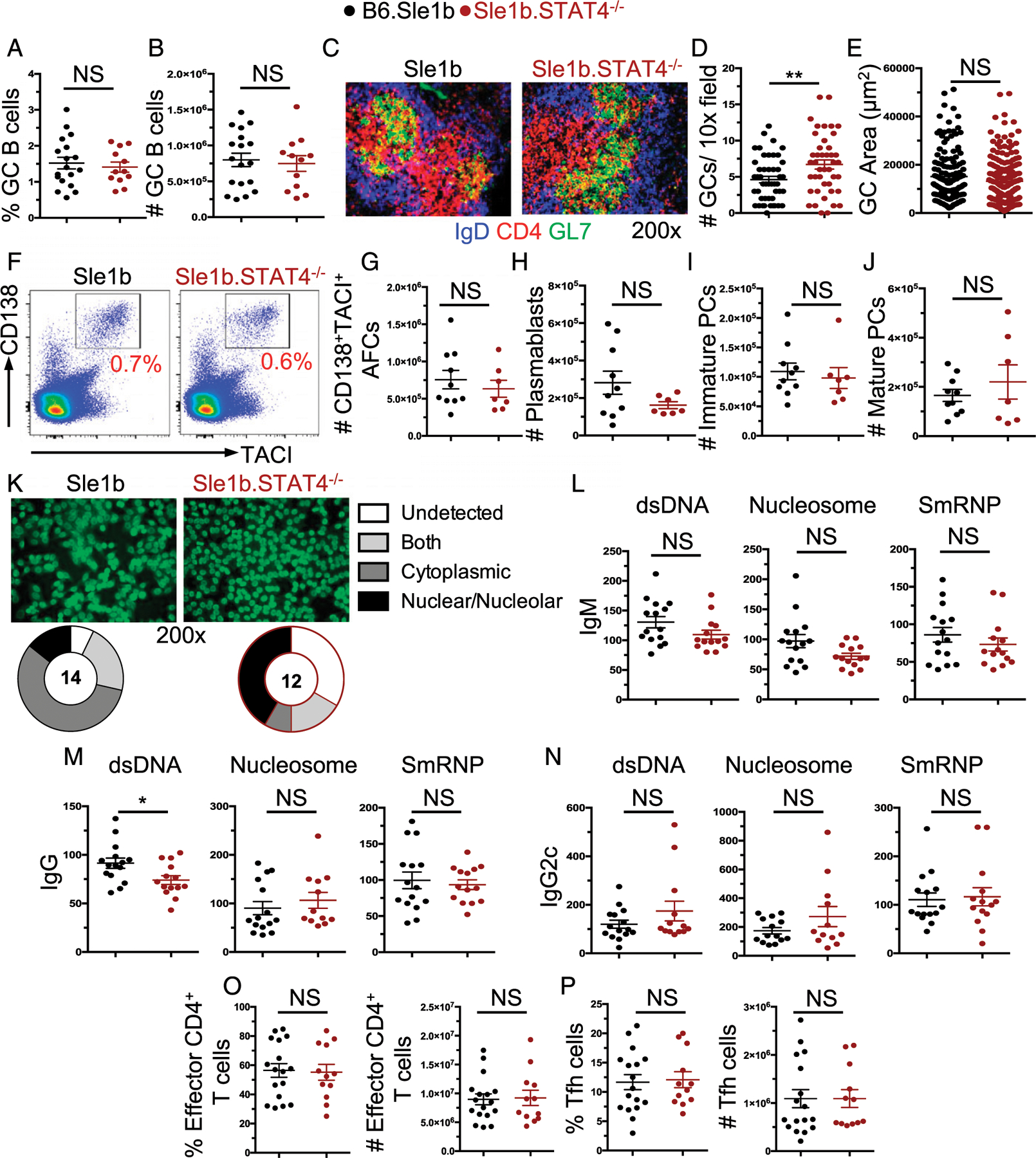
Spt autoimmune responses in Sle1b mice are unaffected by STAT4 deficiency. Spt autoimmune B and T responses were assessed in 4-6-mo-old Sle1b.STAT4^−/−^ and Sle1b control mice. (**A**) Frequency and (**B**) number of GC B cells within the spleens assessed by flow cytometry. (**C**) Representative IF microscopy images of GCs from indicated mice (original magnification ×200). (**D**) Frequency and (**E**) area of GCs within the spleen. (**F**) Representative flow plots and (**G**) number of IgD^−^CD138^+^TACI^+^ AFCs within the spleen. Number of (**H**) plasmablasts, and (**I**) immature plasma cells (PCs) and (**J**) mature/resting PCs within the spleen (57). (**K**) Representative Hep-2 slide analysis of ANA seropositivity and quantification of staining pattern in which the number indicates the number of mice assessed. (**L**) IgM, (**M**) IgG, and (**N**) IgG2c serum autoantibody titers directed against dsDNA, nucleosome, or SmRNP. Frequency and number of (**O**) CD4^+^CD44^+^CD62L^−^ effector T cells and (**P**) CD4^+^CD44^+^CD62L^−^PD-1^+^CXCR5^+^ Tfh within the spleen. Each point represents an individual mouse. *n* = 12–18 mice per genotype. These data represent three independent experiments. *p < 0.05, **p < 0.001.

To exclude a model-specific effect of STAT4 on the regulation of autoimmune GC, AFC, and Th cell responses, we crossed STAT4^−/−^ mice to the autoimmune-prone B6.*Sle1b* mice (Sle1b.STAT4^−/−^). B6.*Sle1b* (*Sle1b*) mice develop heightened Spt-GC and -AFC and Tfh responses resulting in autoantibody production without causing SLE-like pathology (4, 42). Thus, this model allows investigation of the role of STAT4 in the regulation of Spt-AFC and -GC and Tfh responses in the absence of disease. Importantly, autoimmune AFC, GC, and Tfh responses in Sle1b mice were shown to be dependent on IFN-γ signaling, the major Th1-associated cytokine (24). We analyzed 4-6-mo-old mice and found no significant defect in the GC responses in the absence of STAT4 (Fig. 3A–E). In fact, the number of GCs within the spleen were slightly elevated when examined by immunofluorescent (IF) microscopy (Fig. 3D). Further, we observed no deficiency in the AFC responses in the absence of STAT4 (Fig. 3F–J). To determine if the absence of STAT4 may influence the ability of B cells to produce class-switched autoantibodies, we measured serum IgM, IgG, and IgG2c autoantibodies against dsDNA, nucleosome, and SmRNP. Again, we found no significant effect on the ability of Sle1b.STAT4^−/−^ mice to produce autoantibodies of any subtype, with the exception of anti-dsDNA IgG titers, which were only modestly reduced in Sle1b.STAT4^−/−^ mice (Fig. 3L–N). Of note, IgG1, IgG2b, and IgG3 autoantibody titers were also measured and found not to be affected by STAT4 deficiency (data not shown). Hep-2 analysis on the serum of Sle1b and Sle1b.STAT4^−/−^ mice revealed similar ANA seropositivity with some changes in the staining patterns in the absence of STAT4 (Fig. 3K).

We then assessed the T cell compartment in Sle1b.STAT4^−/−^ mice at 4–6 mo of age and again found that effector (CD4^+^CD44^+^CD62L^−^) and follicular (CD4^+^CD44^+^CD62L^−^PD-1^+^CXCR5^+^) T cell populations were intact in STAT4-deficient Sle1b mice (Fig. 3O, 3P). Together, these findings highlight in a second model that STAT4 is not required for Spt-AFC and -GC and Th cell responses and autoantibody production.

### STAT4 is dispensable for TLR7-accelerated autoimmune responses

**FIGURE 4.**
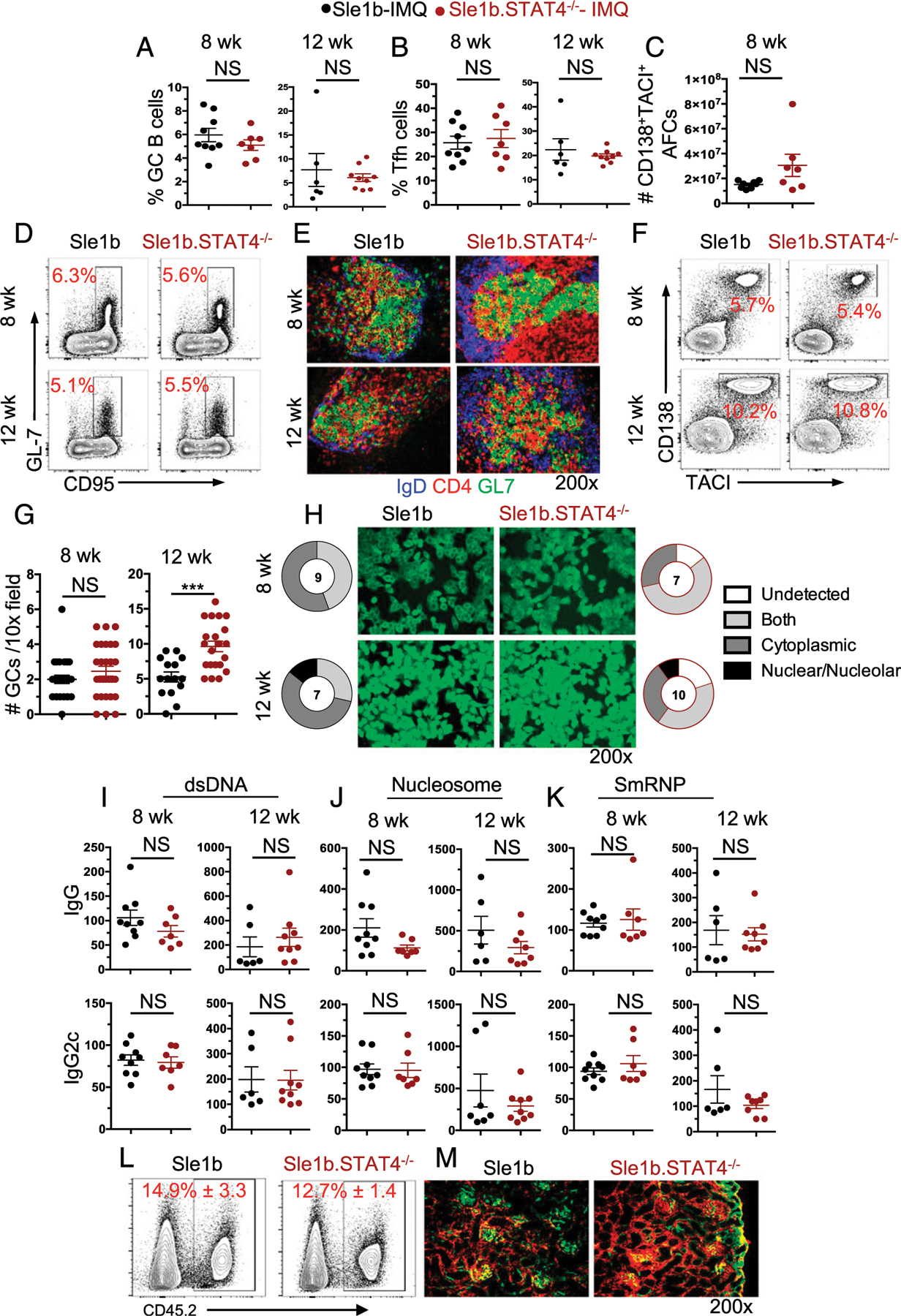
STAT4 is not required for TLR7-driven autoimmune responses and kidney manifestations. Sle1b.STAT4^−/−^ mice and Sle1b mice were treated with the TLR7 ligand Imq epicutaneously three times per week for 8 or 12 wk for the analysis of autoimmune responses and kidney manifestations. Frequency of (**A**) GC B cells pregated on B220^+^ cells and (**B**) Tfh pregated on CD4^+^ cells at 8 and 12 wk after Imq treatment. (**C**) Number of IgD^−^CD138^+^TACI^+^ AFCs within the spleen 8 wk after Imq treatment. (**D**) Representative FACS plots of GC B cells from indicated mice. (**E**) Representative IF microscopy images of GCs from indicated mice. (**F**) Representative FACS plots of AFCs from indicated mice. (**G**) Number of GCs assessed by IF microscopy within the spleens of noted mice. (**H**) Hep-2 slide analysis for ANA seropositivity and pattern assessment from indicated mice in which the number indicates the number of mice assessed per group. Serum IgG and IgG2c autoantibody titers against dsDNA (**I**), nucleosome (**J**), and SmRNP (**K**) were assessed in these mice. (**L**) Representative FACS plots of immune cell infiltration in kidneys. (**M**) Representative images of IC deposition and C3 fluorescence intensity within the kidneys of mice treated with Imq for 12 wk. Each point is an individual mouse. *n* = 7–9 mice per genotype. These data represent three independent experiments. ***p < 0.0001.

Although Sle1b mice develop mild autoimmune responses without causing disease, we recently have shown that mild autoimmune responses in Sle1b mice could be accelerated by treating these mice epicutaneously with TLR7 agonist Imq, promoting the development of SLE-like disease (4). In this context, mice can be treated with Imq for 8 wk to drive heightened AFC, GC, Tfh, and autoantibody responses and for 12 wk to promote significant IC deposition and pathology within the kidneys (4). Of note, neither treatment duration leads to any visible damage or swelling of the ear, eliminating the concern of pathogen introduction through wounded tissue (4). Importantly, a deficiency in global or B cell–intrinsic IFN-γ signaling was shown to ablate autoimmune responses and SLE pathogenesis, and Imq treatment led to robust IFN-γ production by Tfh (4). To explore the involvement of STAT4 in TLR7-accelerated AFC, GC, and Tfh responses and SLE manifestations, Sle1b and Sle1b.STAT4^−/−^ mice were treated with Imq for either 8 or 12 wk. Again, in the absence of STAT4, we did not appreciate any deficits in GC (Fig. 4A, 4D, 4E, and 4G), Tfh (Fig. 4B), AFC (Fig. 4C, 4F), and CD4^+^ T effector cell (data not shown) responses at either time point assessed. We also did not find any significant difference in ANA seropositivity and IgG or IgG2c autoantibody titers (Fig. 4H–K). Further, we analyzed immune cell infiltration and IC deposition in the kidneys of mice treated with Imq for 12 wk and found no significant difference in the absence of STAT4 (Fig. 4L, 4M). Treatment of B6 and STAT4^−/−^ mice with Imq for 5 wk also yielded similar B and T cell responses (data not shown). Together, these findings highlight a surprising lack of STAT4 requirement in TLR7-accelerated AFC, GC, and Tfh responses and SLE manifestations.

### Foreign Ag–driven AFC, GC, and Th cell responses are largely unattenuated in the absence of STAT4

**FIGURE 5.**
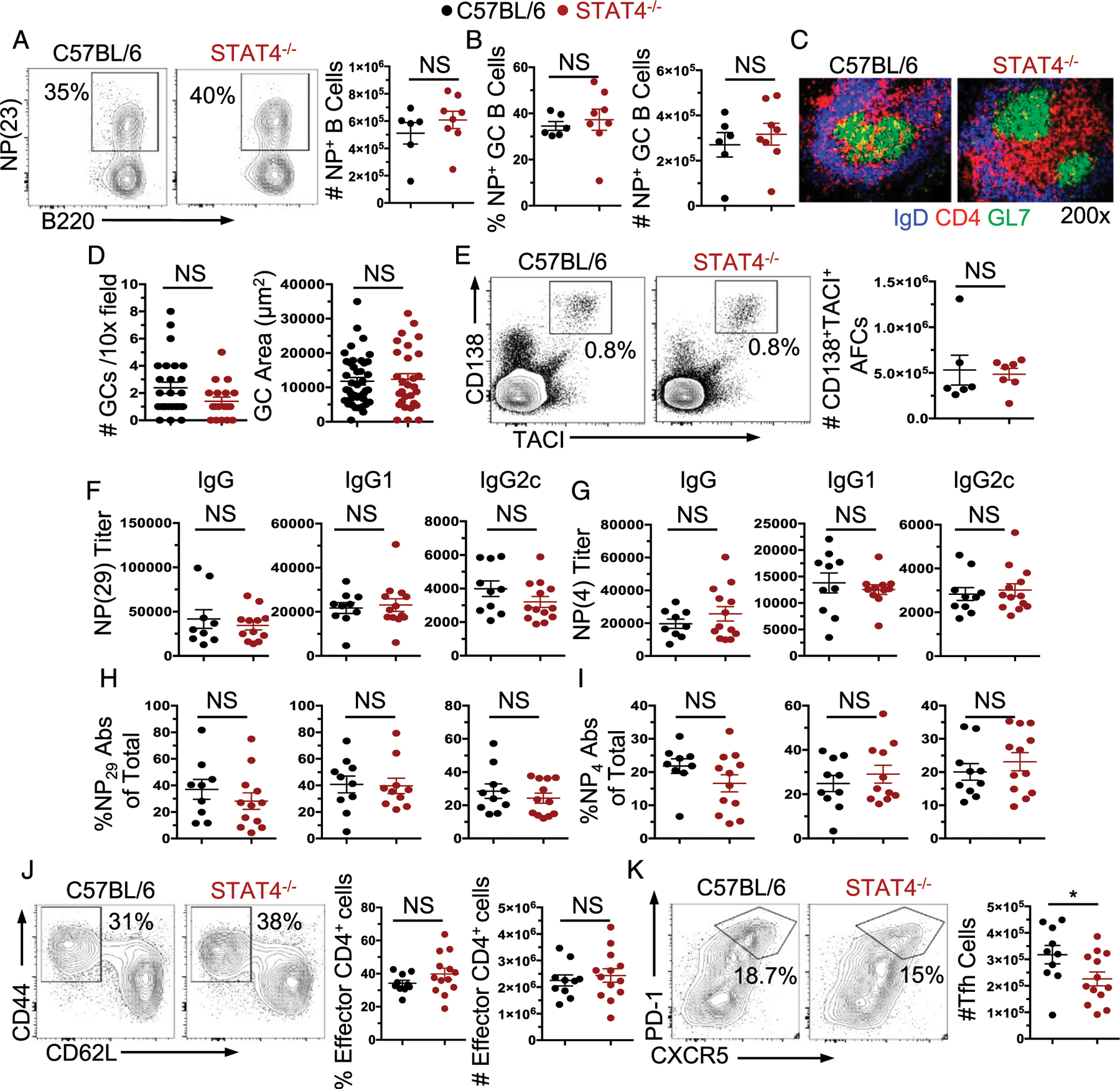
Immunization-induced B cell responses do not require STAT4. C57BL/6 and STAT4^−/−^ mice were immunized with NP-KLH in CFA, boosted on day 7 after primary immunization with NP-KLH in IFA, and the response was assessed on day 14 after primary immunization. The frequency and number of NP-specific (**A**) total and (**B**) GC B cells within the spleens of immunized mice. (**C**) Representative IF microscopy images of IgD^−^GL7^+^ GCs from the spleens of immunized mice. (**D**) Quantification of the number and area of GCs within the spleens of immunized mice. (**E**) Representative FACS plots of frequency and number of IgD^−^CD138^+^TACI^+^ AFCs within the spleens of immunized mice. (**F**) The low-affinity (NP_29_) and (**G**) high-affinity (NP_4_) serum IgG, IgG1, and IgG2c Ab titers in immunized mice. The percentage of NP-specific Ab titers relative to total amount of each isotype determined for NP_29_ (**H**)– and NP_4_ (**I**)–specific serum IgG, IgG1, and IgG2c Abs. Each point is an individual mouse. Representative FACS plots and the frequency and number of (**J**) CD4^+^CD44^+^CD62L^−^ effector T cells and (**K**) CD4^+^CD44^+^CD62L^−^PD-1^+^CXCR5^+^ Tfh within the spleen following immunization. *n* = 10–13 mice per genotype, and data represent three independent experiments. *p < 0.05.

The role of STAT4 in foreign Ag–induced AFC, GC, and Th cell responses remains unclear. Based on our findings demonstrating a lack of necessity for STAT4 in the development of an autoimmune response, we asked whether STAT4 would be important for AFC, GC, and Th cell responses to exogenous Ag. We immunized B6 and STAT4^−/−^ mice with NP-KLH in CFA and analyzed the response at day 14 postimmunization. Consistent with our findings in autoimmune-prone mice, we found that both total and NP-specific GC B cell responses were intact in STAT4^−/−^ mice (Fig. 5A–D). We then assessed the AFC/plasma cell compartment in these mice and observed that total (Fig. 5E) and subpopulations of AFC (plasmablast, immature plasma cell, and mature plasma cell; data not shown) responses were all intact in the absence of STAT4. We also found no impact of STAT4 deficiency on high-affinity (NP4) and low-affinity (NP29) IgG, IgG1, and IgG2c serum Ab titers (Fig. 5F, 5G). We then measured T cell responses in these mice and found that the frequency and number of effector CD4^+^ T cells were not affected by the lack of STAT4 (Fig. 5J), although the frequency and number of Tfh were slightly reduced in STAT4^−/−^ mice (Fig. 5K). In total, similar to autoimmune AFC and GC responses in SLE-prone mice, these data demonstrate a minor role of STAT4 in foreign Ag–driven AFC, GC, and Th cell responses.

### Pathogen-induced AFC, GC, and Th cell responses are intact in STAT4^−/−^ mice during influenza virus infection

**FIGURE 6.**
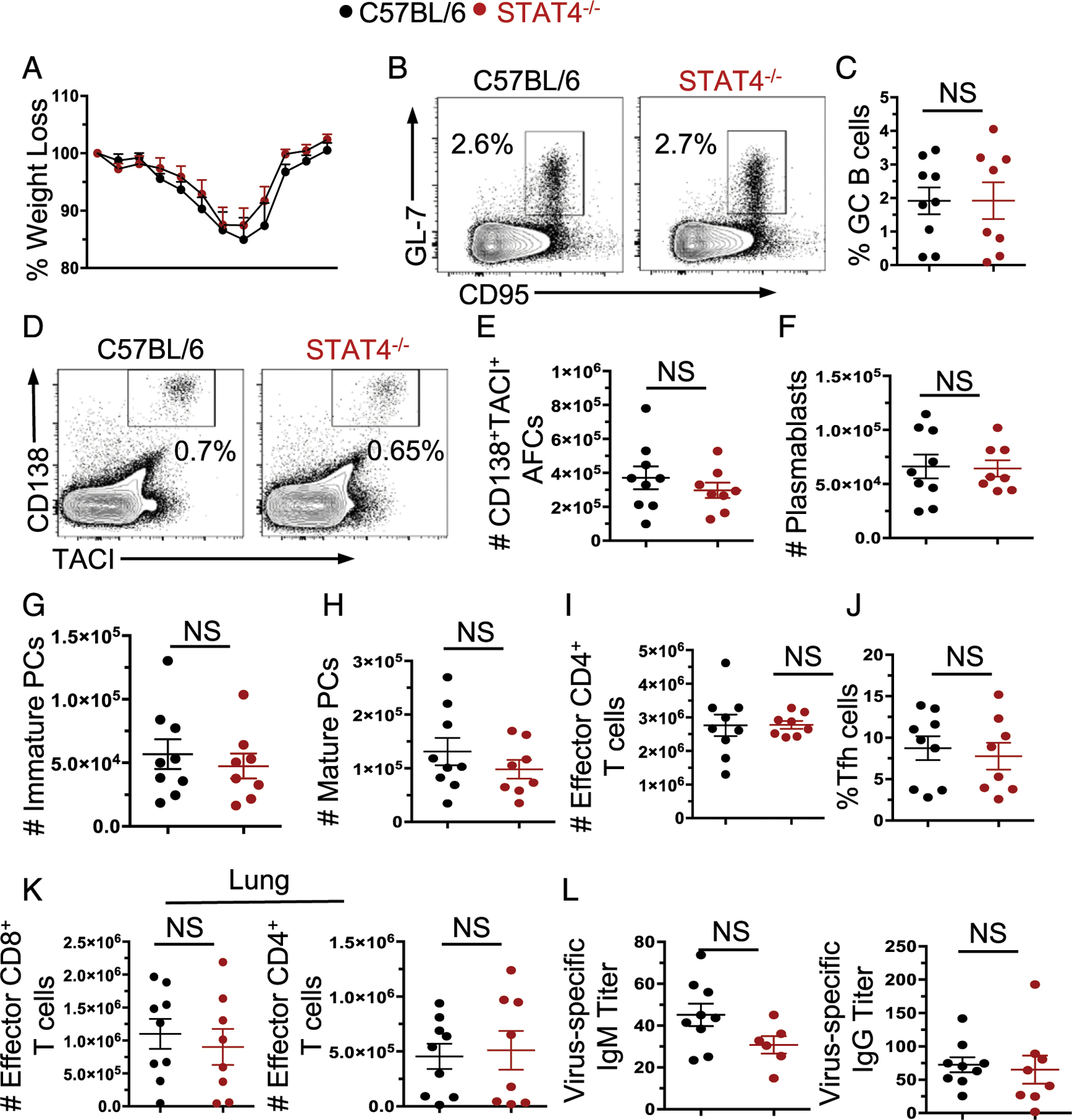
B cell responses to influenza viral infection are intact in the absence of STAT4. C57BL/6 and STAT4^−/−^ mice were infected intranasally with 1000 fluorescent focus counts of influenza virus and the influenza virus driven response was assessed at day 14 postinfection. (**A**) Weight loss percentage after influenza virus infection. (**B**) Representative FACS plots and (**C**) the frequency of GC B cells within the spleen of influenza virus–infected mice. (**D**) Representative FACS plots and the number of (**E**) IgD^−^CD138^+^TACI^+^ AFCs, (**F**) plasmablasts, and (**G**) immature and (**H**) mature plasma cells (PCs) within the spleens of infected mice. (**I**) Number of CD4^+^CD44^+^CD62L^−^ effector T cells within the spleen of infected mice. (**J**) Frequency of Tfh within the spleen. (**K**) The number of CD8a^+^CD44^+^CD62L^−^ and CD4^+^CD44^+^CD62L^−^ effector T cells within the lung. (**L**) Influenza virus–specific serum IgM and IgG titers were assessed. Each point is an individual mouse. *n* = 8–9 mice per genotype, two independent experiments.

Given that protein immunization does not completely replicate a pathogen-driven response, we infected STAT4^−/−^ and B6 control mice with influenza virus intranasally and harvested the spleens and lungs on day 14 postinfection. Weight loss through the course of the infection was not different between STAT4^−/−^ and B6 control mice (Fig. 6A). Within the spleens, we identified no significant differences in GCB cell (Fig. 6B, 6C) and AFCresponses (Fig. 6D–H). We also did not observe differences in effector CD4^+^ T cell (Fig. 6I) or Tfh responses (Fig. 6J). We further identified no impact of STAT4 deficiency on short-lived effector cell/memory precursor effector cell CD4^+^ T cell populations within the spleens or lungs (data not shown). We then characterized the immune cell infiltrates into the lung and again found no differences in effector CD8^+^ T cell or CD4^+^ T cell responses (Fig. 6K) within the lung. Further, we detected no significant differences in influenza virus–specific serum IgM or IgG titers between the strains (Fig. 6L). Together, these findings paired with the protein immunization data highlight that STAT4 is also largely dispensable for exogenous Ag–driven AFC, GC, and Th cell responses, which confer protection.

## DISCUSSION

Despite the important role for STAT4 in SLE autoimmunity based on GWAS and mouse studies (26–31, 33, 47, 48), how STAT4 may regulate autoimmune AFC, GC, and Tfh responses in SLE was previously not explored. The goal of the current manuscript was to determine the effects of STAT4 deficiency on AFC, GC, and Tfh responses in autoimmune- and SLE-prone mouse models. To our surprise, we found STAT4 to be largely dispensable for promoting AFC, GC, and Tfh responses in three different autoimmune- and SLE-prone mouse models. Consistent with no effects of STAT4 on the regulation of autoimmune AFC, GC, and Tfh responses, autoantibody production and IC deposition and immune cell infiltration in the kidney were largely unaffected in autoimmune- and SLE-prone mice deficient in STAT4. We further determined the role of STAT4 in the regulation of AFC, GC, and Tfh during foreign Ag–driven responses such as NP-KLH immunization or influenza viral infection. We also found STAT4 to be not required for such foreign Ag–driven responses. We further confirmed that AFC, GC, and Tfh responses in STAT4-deficient mice were observed in the absence of any detectable STAT4 protein. Together, our data preclude a role for STAT4 in SLE manifestations in Spt FcγRIIB^−/−^ and Sle1b and TLR7-induced Sle1b models and in promoting AFC, GC, and Tfh responses in autoimmune and foreign Ag–driven responses, including influenza viral infection. Whether there is any possibility for a truncated STAT4 protein to be functional in this newly generated, CRISPR-based, and commercially available STAT4^−/−^ mice needs to be further investigated using Abs targeting different epitopes of STAT4.

Early studies established a strong role for STAT4 signaling in differentiating CD4^+^ T cells toward a Th1 lineage–producing IFN-γ (11, 16, 17, 45, 49–52). A more recent in vitro study highlighted the role for STAT4 in inducing IL-21 and Bcl6 genes in T cells, promoting the differentiation of T cells with features of Tfh producing both IL21 and IFN-γ (19). A recent in vivo study showed that lymphocytic choriomeningitis virus–infected STAT4^−/−^ mice developed intact CD4^+^ Th1 and Tfh responses, although phosphorylation of STAT4 was required for optimal IFN-γ production by Th1 cells (20). In the current study, we found that the expression of Tbet by Th1 cells and IFN-γ in splenocytes from STAT4-deficient FcγRIIB^−/−^ mice were unaltered. Interestingly, we previously highlighted the importance of Tbet and IFN-γ signaling in SLE-associated AFC, GC, and Tfh responses and SLE disease development in TLR7-induced Sle1b model (4, 24). In contrast, we found that STAT4 was not required for AFC, GC, and Tfh responses and autoimmunity development in this SLE model. Together, our current and published data suggest STAT4-independent and IFN-γ–STAT1–Tbet-dependent development of SLE-associated AFC, GC, and Tfh responses in SLE-prone mice. Whether the previously established role of STAT4 in differentiating CD4^+^ T cells into Th1 or Tfh-like cells in vitro can be recapitulated using current STAT4^−/−^ mice needs to be further investigated.

STAT4 expression is elevated in certain populations of SLE patients, and risk variants in the *STAT4* gene are common findings in the GWAS of SLE patients (26, 32). However, to our knowledge, no one has definitively demonstrated the effects of individual STAT4 risk alleles on autoimmunity development or cellular functions beyond association. This is primarily because these risk variants are located in the intronic region of the *STAT4* gene, and a number of risk SNPs are in genetic linkage disequilibrium, and no mouse model could be developed to definitively examine the effects of individual SNPs. Although we used the knockout systems to identify the role of STAT4 in autoimmune responses and autoimmunity development in autoimmune- or SLE-prone mouse models, it is possible that overexpression of STAT4 in SLE patients may take on novel functions or may function in concert with other genetic predispositions, such as IRF5 variants, to contribute to SLE disease (47). The *STAT4* and *STAT1* genes are located next to each other on human chromosome 2 (48). A previous fine-mapping study of the *STAT1*–*STAT4* locus indicated that the risk allele of rs11889341 led to elevated STAT1, but not STAT4, expression in B cells (48). This could at least partially explain why an STAT4 deletion in our study had no effects on autoimmune B cell responses and autoimmunity development in several autoimmune- or SLE-prone mice, whereas an STAT1 deficiency ablated such responses (4, 24, 42). Given the established role of IL-12–driven STAT4 signaling in promoting human Tfh development (53, 54) and a strong association of STAT4 risk variants with SLE, it is also possible that mouse and human STAT4 differentially function to promote B and T cell responses in SLE.

Combining our current data from the Sle1b and FcγRIIB^−/−^ models, which are on B6 backgrounds, with previous studies that used polygenic models (34, 35) may indicate a model-dependent role of STAT4 in autoimmune responses and SLE manifestations. The NZM2328 mouse model deficient in STAT4 had reduced autoantibody production but increased kidney disease and mortality, whereas STAT4 deficiency in the SLE 1,2,3 triple congenic model resulted in reduced responses in various autoimmune parameters such as autoantibody production, kidney disease, and mortality (34, 35). We, however, found no or minor effects of STAT4 on AFC, GC, and Tfh responses and IC deposition and immune cell infiltration in the kidney of Sle1b and FcγRIIB^−/−^ mice. One possibility for differential outcomes between our and previous studies could be that STAT4 interacts with other autoimmune genes in NZM2328 and 1,2,3 triple congenic mice to promote autoimmune responses and disease manifestations. Further, it should be noted that STAT4^−/−^ mice used in our study are different from those in previous studies. Previous studies crossed STAT4^−/−^ mice generated on a BALB/c or B6.129 mixed background to SLE-prone mice (17, 34, 35), whereas STAT4^−/−^ mice we used are commercially available and were generated directly on a B6 background, eliminating the role of background genes in our current findings. Thus, it is possible that residual genes from the BALB/c or B6.129 background may be responsible for the differential outcomes. Future work will be required to identify the role of background genes in resolving the discrepancy between our current findings and previously published data.

Earlier studies suggested that immunization of STAT4^−/−^ mice resulted in a reduced capacity for T cells to produce IFN-γ paired with an increase in Th2-driven responses associated with the production of IL-4, IL-5, and IL-10 (12). With a shift from Th1- to Th2-driven responses, STAT4^−/−^ mice were shown to have a shift in Ag-specific Ab production from IgG2a to IgG1 (12). We, however, did not find differences in AFC, GC, and Th cell responses as well as high- and low-affinity, Ag-specific IgG, IgG1, and IgG2c Abs in the absence of STAT4. Similar results were observed with influenza virus infection. Discrepancy in class-switched IgG Ab responses between published and our current data are not clear. As described above, the contributions of background genes to differential outcomes in Ab responses between earlier studies and the current study cannot be ruled out. Further, in autoimmune models of experimental autoimmune encephalomyelitis, arthritis, colitis, myocarditis, and diabetes in which T cells may play a significant role, STAT4 deficiency demonstrated a reduction in autoimmune manifestations associated with reduced IFN-γ production and a switch from a dominant IgG2a response to IgG1 (12). Of note, STAT4^−/−^ mice developed equivalent susceptibility in mouse models of myasthenia gravis and Graves disease, both of which are mediated by Ab-dependent mechanisms (55, 56). Together, these data suggest model-dependent effects of STAT4 in various autoimmune diseases and foreign Ag–driven responses in which STAT4 contributes to T cell–mediated autoimmunity, but it is largely dispensable for AFC, GC, and Th cell responses in B cell–mediated autoimmunity, including SLE and certain foreign Ag–driven responses.

Overall, using STAT4-deficient mice recently developed on a B6 background, our study highlights that STAT4 is not required for foreign Ag–driven AFC, GC, and Th cell responses and Ag-specific Ab production during protein immunization or influenza virus infection. STAT4 is also largely dispensable for the regulation of autoimmune AFC, GC, and Th cell responses, autoantibody production and SLE manifestations in Spt autoimmune-prone Sle1b and SLE-prone FcγRIIB^−/−^ mice, and TLR7-induced, SLE-prone Sle1b mice. Whether background genes in STAT4^−/−^ mice or polygenic SLE models contribute to differential outcomes between our study and previous studies in identifying the role of STAT4 in promoting autoimmune responses and SLE autoimmunity remains to be resolved. Given published data showing an indispensable role of IFN-γ–STAT1–Tbet signaling in the development of autoimmune AFC and GC responses and autoimmunity in SLE-prone mice (4, 24, 25, 42) and regulation of STAT1 in B cells by the *STAT4* risk allele of rs11889341 (48), it is tempting to speculate whether *STAT1*, and not *STAT4*, is the culprit gene in promoting SLE autoimmunity in patients.

## Supplementary Material

Figure S1

## Abbreviations used in this article:

AFC: Ab-forming cell
ANA: antinuclear Ab
GC: germinal center
GWAS: genome-wide association study
IC: immune complex
IF: immunofluorescent
Imq: imiquimod
NP-KLH: 4-hydroxy-3-nitrophenol–keyhole limpet hemocyanin
SmRNP: small ribonucleoprotein
Spt: spontaneous
Tfh: T follicular helper cell
