# Supplementary figures and images for: STAT4 Is Largely Dispensable for Systemic Lupus Erythematosus–like Autoimmune- and Foreign Antigen–Driven Antibody-Forming Cell, Germinal Center, and Follicular Th Cell Responses
Source: Immunohorizons. Author manuscript; Available in PMC 2025 May 20. (PMC12090115; doi:10.4049/immunohorizons.2000111)

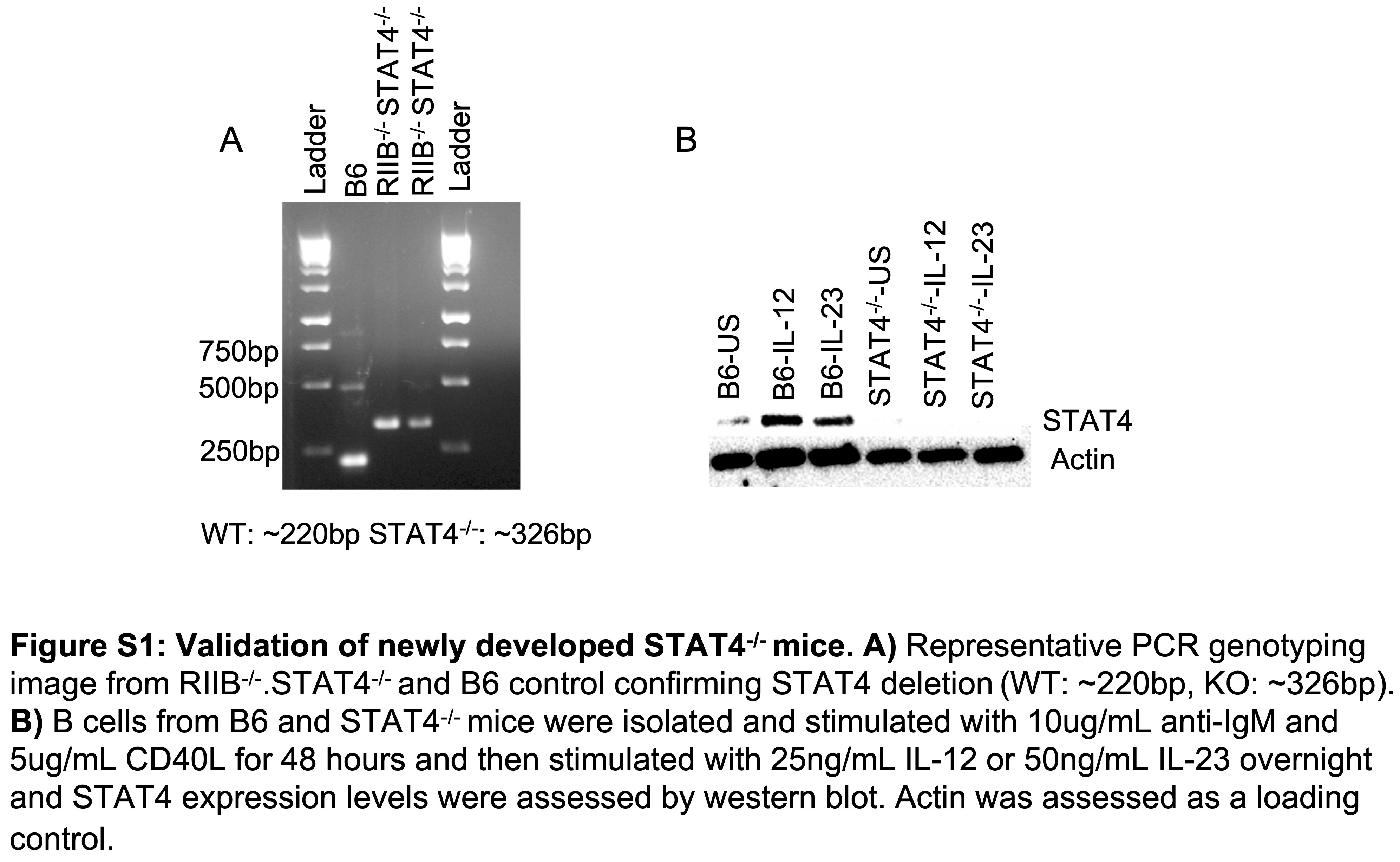

Supplement: Figure S1 [file NIHMS2075542-supplement-Figure_S1.jpg]
